# Circulating exosomes carrying an immunosuppressive cargo interfere with cellular immunotherapy in acute myeloid leukemia

**DOI:** 10.1038/s41598-017-14661-w

**Published:** 2017-10-31

**Authors:** Chang-Sook Hong, Priyanka Sharma, Saigopalakrishna S. Yerneni, Patricia Simms, Edwin K. Jackson, Theresa L. Whiteside, Michael Boyiadzis

**Affiliations:** 1 Department of Pathology, University of Pittsburgh School of Medicine, Pittsburgh, PA USA; 2 Department of Biomedical Engineering, Carnegie Mellon University, Pittsburgh, PA USA; 3 FACS Core Facility, Loyola University School of Medicine, Maywood, IL 60153 USA; 4 Department of Pharmacology and Chemical Biology, University of Pittsburgh School of Medicine, Pittsburgh, PA USA; 5 Departments of Immunology and Otolaryngology, University of Pittsburgh School of Medicine, Pittsburgh, PA USA; 6 UPMC Hillman Cancer Center, Pittsburgh, PA USA; 7 Department of Medicine, Division of hematology-Oncology, University of Pittsburgh School of Medicine, Pittsburgh, PA USA

## Abstract

Exosomes, small (30–150 nm) extracellular vesicles (EVs) isolated from plasma of patients with acute myeloid leukemia (AML) carry leukemia-associated antigens and multiple inhibitory molecules. Circulating exosomes can deliver suppressive cargos to immune recipient cells, inhibiting anti-tumor activities. Pre-therapy plasma of refractory/relapsed AML patients contains elevated levels of immunosuppressive exosomes which interfere with anti-leukemia functions of activated immune cells. We show that exosomes isolated from pre-therapy plasma of the AML patients receiving adoptive NK-92 cell therapy block anti-leukemia cytotoxicity of NK-92 cells and other NK-92 cell functions. NK-92 cells do not internalize AML exosomes. Instead, signaling via surface receptors expressed on NK-92 cells, AML exosomes simultaneously deliver multiple inhibitory ligands to the cognate receptors. The signals are processed downstream and activate multiple suppressive pathways in NK-92 cells. AML exosomes reprogram NK-92 cells, interfering with their anti-leukemia functions and reducing the therapeutic potential of adoptive cell transfers. Plasma-derived exosomes interfere with immune cells used for adoptive cell therapy and may limit expected therapeutic benefits of adoptive cell therapy.

## Introduction

Adoptive cell therapy (ACT), including transfer of activated NK cells, is currently under active investigation for patients with refractory/relapsed acute myeloid leukemia (AML). Administration of ACT to AML patients is based on the rationale that adoptively- transferred NK cells will eliminate leukemic blasts in the periphery as well as in the bone marrow and will promote recovery of anti-leukemia immunity compromised by the progressing disease and/or chemotherapy^1–3^. Immunological dysfunction in patients with AML, including deficits in NK-cell numbers and activity, elevation in the number of circulating regulatory T cells (Treg) and dysregulation in the cytokine profiles could contribute to leukemia relapse^4–7^.

In hope of restoring, at least in part, anti-leukemia immunity in patients with relapsed/refractory AML, we recently completed a phase 1 clinical trial of ACT with NK-92 cells (a human IL-2 dependent NK-cell line FDA-approved for human ACT)^8^.

The ACT was well tolerated, but no immunological recovery and no complete responces^8^. These disappointing results could be attributed to profoundly immunosuppressive microenvironment in relapsed/refractor AML patients. Among many potential mechanisms responsible for impaired anti-leukemia activity in AML that could also interfere with adoptively transferred NK-92 cells is exosome-mediated immune suppression^9^.

Exosomes are the smallest (30–150 mm) of extracellular vesicles (EVs) circulating freely throughout the body and serving as an efficient communication system^9–11^. We have reported that blast-derived exosomes carrying immunosuppressive cargos accumulate in plasma of AML patients and include dysfunction of immune cells^12–14^. The pre-ACT levels of plasma-derived exosomes were highly elevated in the patients enrolled in the trial. Therefore, we hypothesized that NK-92 cells transferred into the environment dominated by immunosuppressive exosomes failed to mediate anti-leukemia activity. To test the hypothesis, we isolated exosomes from the pre-therapy plasma specimens of AML patients enrolled in the trial and studied their effects on NK-92 cell functions. We show that exosomes isolated from pre-therapy plasma of these patients inhibited various NK-92 cell functions *ex vivo* and interfered with anti-leukemia activity of these cells. Further, the *ex vivo* blockade of exosome-mediated suppression in part restored NK-92 cell functions. These results suggest that in malignancy, plasma-derived exosomes can interfere with immune cells used for ACT and may limit expected therapeutic benefits of ACT.

## Results

### Characterization of AML exosomes

**Figure 1:**
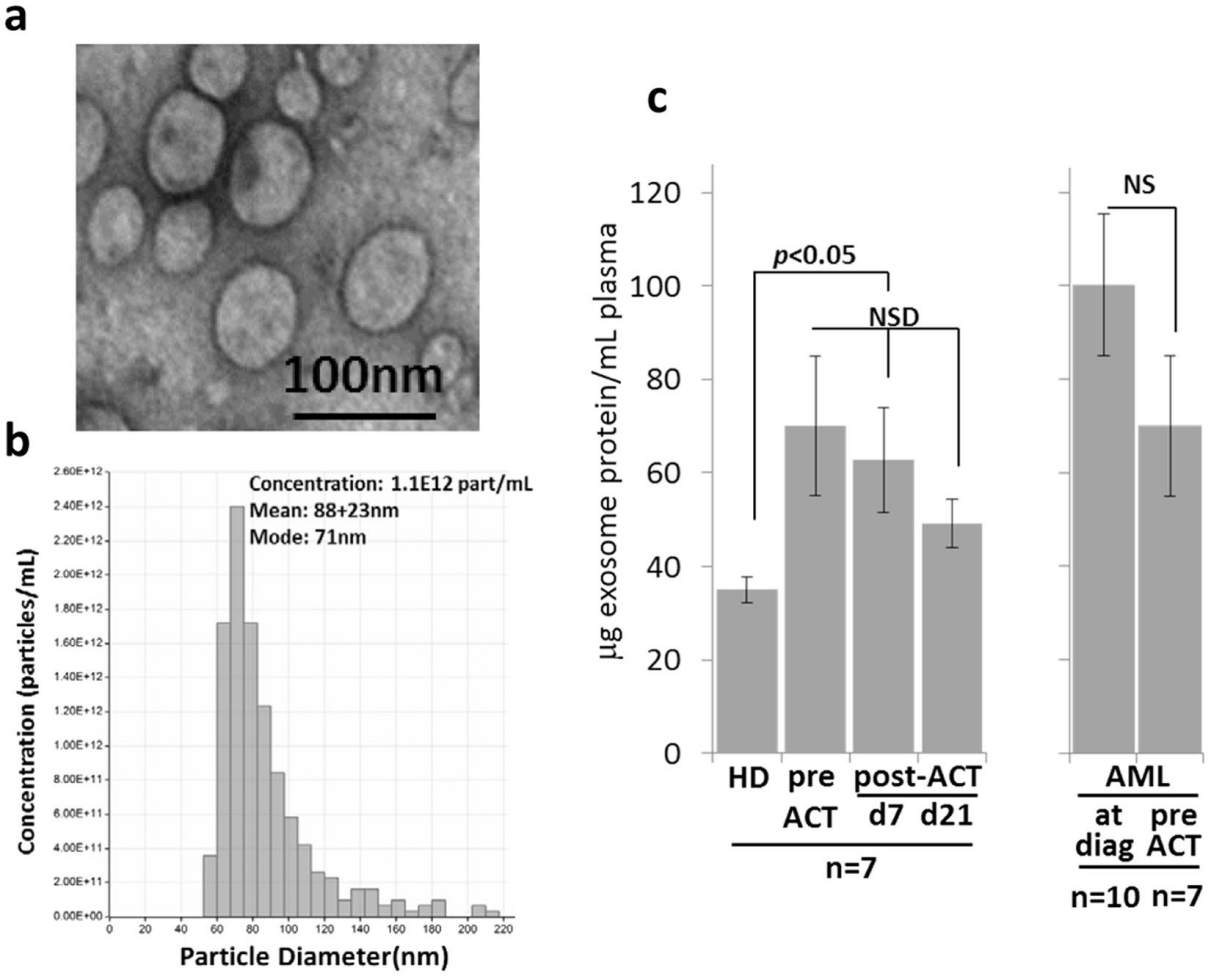
Characteristics and plasma levels of AML exosomes. (**a**) Transmission electron microscopy of isolated AML exosomes. (**b**) Size and concentration of AML exosomes as determined by tunable resistive sensing (TRPS). (**c**) Protein levels (in µg/mL plasma) of exosomes isolated from plasma of normal donors (ND), AML patients pre-ACT or post ACT(on days7 or 21) and of random AML patients at diagnosis vs AML patients prior to ACT.

**Figure 2:**
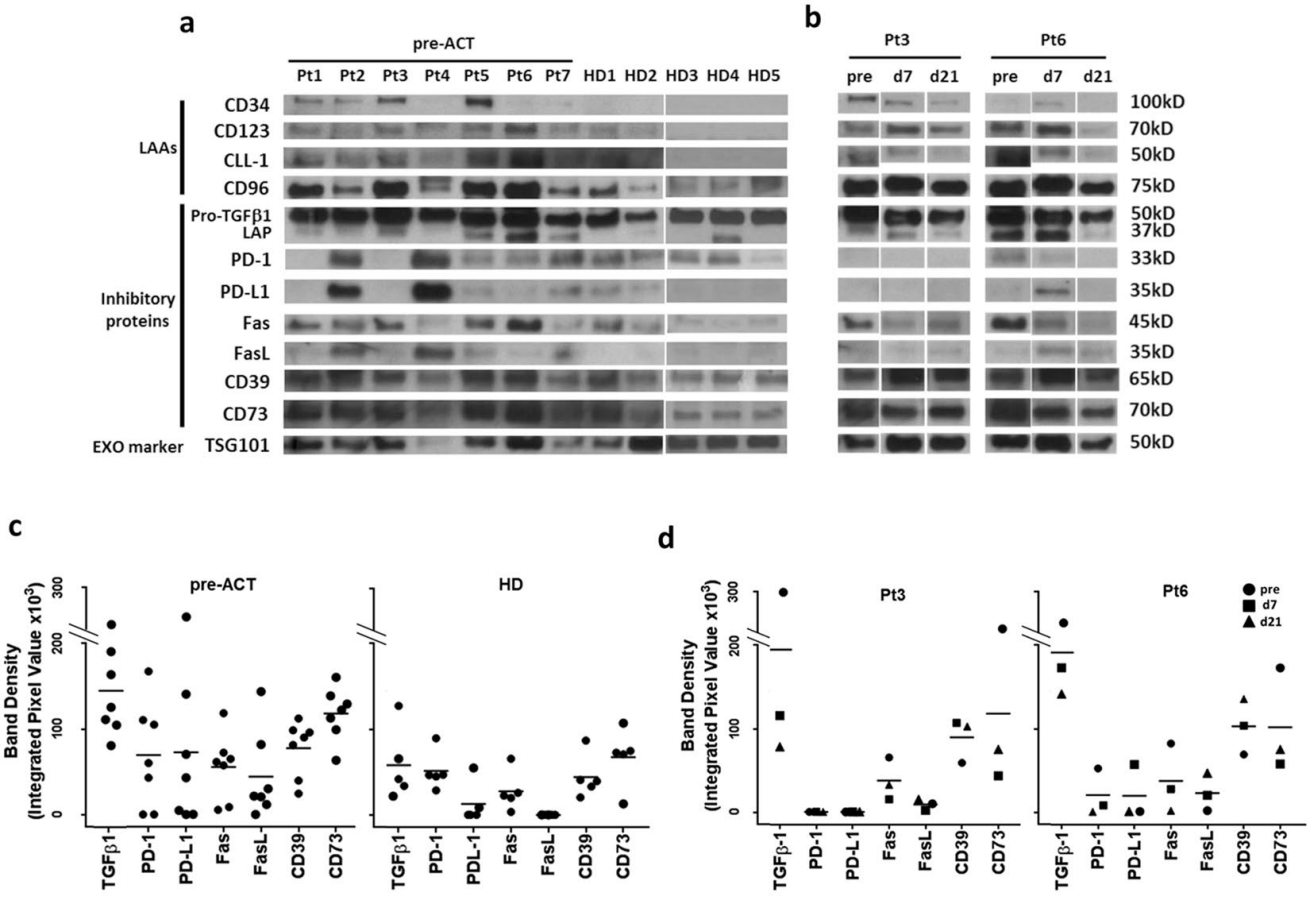
Molecular profiles of AML exosomes. (**a**) Western blots of exosomes isolated from plasma of 7AML patients prior to ACT or in (**b**). post ACT (day7 and 21, pts #3 and #6) or from plasma of 5 HDs. The blots for each patient or HD are from different gels, as indicated by spaces between the blots. (**c**) and (**d**). Semi-quantitative densitometry for the Western blots shown in (**a** and **b**). Band density was determined as described in Methods.

Transmission electron microscopy of exosomes isolated from pre-therapy plasma of patients with relapsed/refractory AML showed the presence of vesicles sized at 30–150 nm (Fig. 1a,b) and similar to vesicles present in plasma of all other AML patients^14,15^. The mean exosome protein levels were significantly elevated in patients’ versus HD’s plasma and remained persistently elevated following ACT (Fig. 1c). The pre-therapy exosome protein levels in plasma of the 7 AML patients receiving ACT were equally as high (Fig. 1c). The molecular profiles of AML exosomes isolated from pre-therapy plasma were enriched in leukemia associated antigens (LAAs) and in proteins that mediate immune suppression, such as TGF-β1/LAP, CD39/CD73 ectoenzymes, PD1/PD-L1 or Fas/FasL (Fig. 2a). Notably, the exosome protein profiles were distinct for each of the 7 AML patients. In semi-quantitative density analyses of Western blots, AML exosomes carried significantly higher levels of immunoinhibitory proteins than exosomes of HDs (Fig. 2c). Furthermore, the molecular profile of exosomes isolated from AML plasma following ACT on day 7 or 21 remained enriched in immunoinhibitory proteins (Fig. 2b,c,d).

### Effects of AML exosomes on NK-92 cell proliferation and survival

The effect of AML exosomes on NK-92 cell proliferation was examined. In Supplementary Figure 1A, NK-92 cells cultured in X-VIVO medium are seen to form large, well-defined clusters, which shrink in size upon exosome addition, releasing single cells. At higher exosome concentrations, the cell clusters shrink further in size, and numbers of single cells increase. Proliferation of NK-92 cells measured in MTT assays, was significantly decreased upon addition of exosomes (Supplementary Figure 1B). However, NK-92 cells were resistant to apoptosis, did not bind Annexin V and were PI negative following co-incubation with AML exosomes (Supplementary Figure 1C).

### AML exosomes inhibit NKG2D expression and cytolytic activity of NK-92 cells

**Figure 3:**
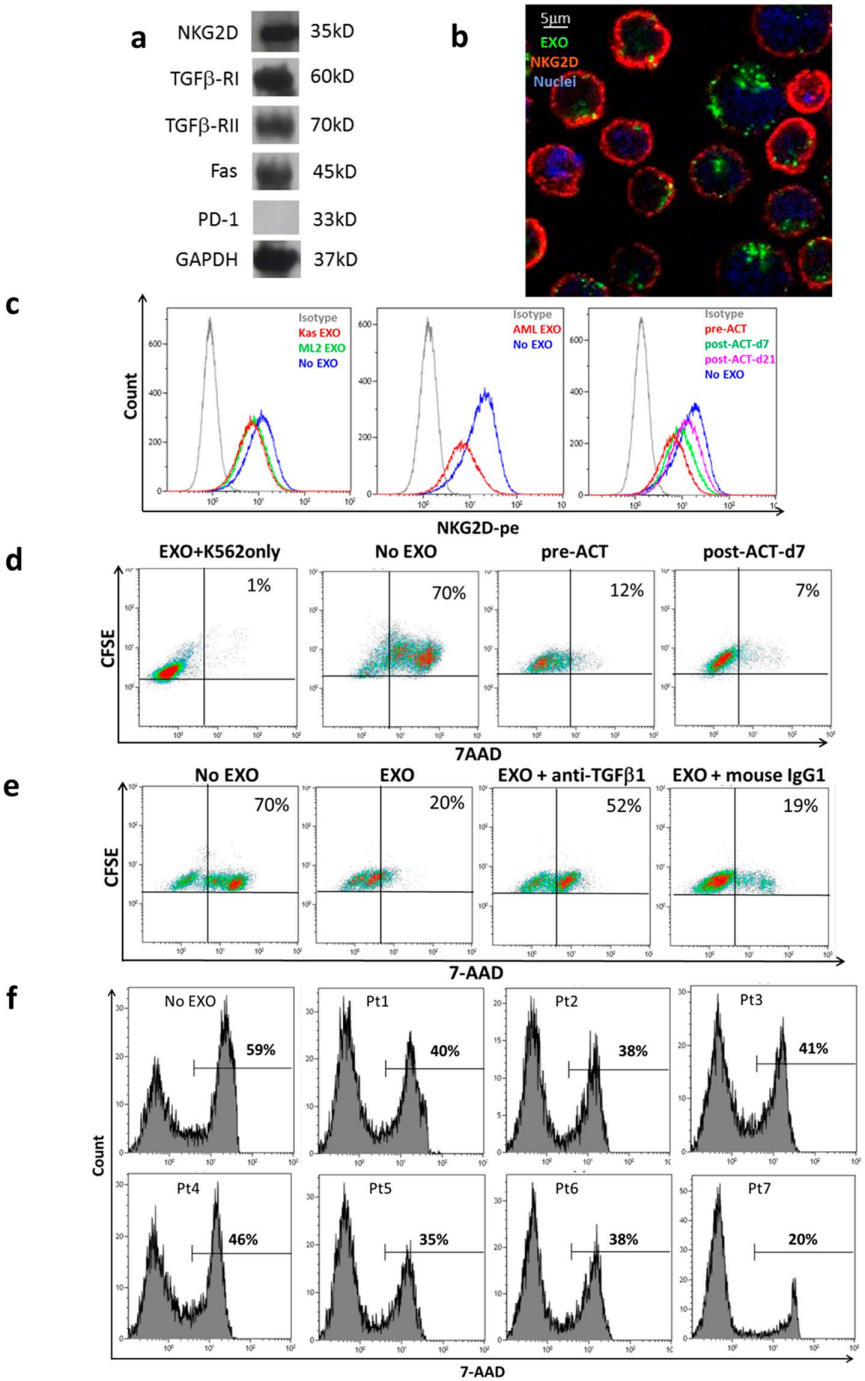
NKG2D receptor downregulation in NK-92 cells and inhibition of NK-92 cytotoxicity by pre-therapy AML exosomes. (**a**) Western blot profiles for selected receptors and ligands on NK-92 cells. Original images of the blots can be found in the Supplementary Information. (**b**) Confocal microscopy for expression of NKG2D (red) on NK-92 cells following 24 h co-incubation with AML exosomes labeled with the PKH67dye (green). Nuclei labeled with the Hoechst dye (blue). NK-92 cells binding exosomes (green) express little or no NKG2D (red). Shown is 1representative of 3 experiments performed with exosomes isolated from plasma of different AML patients (see Table 1). (**c**) Flow cytometry showing downregulation of NKG2D on NK-92 cells co-incubated ± exosomes. Exosomes were isolated from supernatants of AML cell lines (Kasumi-1 or ML2); plasma of an AML patient at diagnosis; from pre-ACT or post ACT (days 7 and 21) plasma of a representative #4 AML patient. Analysis of the data obtained for 4 different AML patients is shown in Table 1. (**d**) Inhibition of cytotoxicity mediated by NK-92 cells against K562 targets, Flow-cytometry-based cytotoxicity assay was performed as described in Methods. The data are for exosomes of patient # 1. The experiment was performed in duplicate using exosomes from 3 AML patients (see Table 2). (**e**) Neutralization with anti TGF-β Ab of exosome-mediated inhibition of cytotoxicity mediated by NK-92 cells. TGFβ+ AML exosomes were pre-incubated with the Ab for 2 h. A representative experiment performed with exosomes isolated from plasma of patient #3. (**f**) Inhibition of NK-92 cytotoxicity against primary AML blasts by exosomes isolated from pre-ACT plasma each AML patient treated with ACT. NK-92 cells were co-incubated ± exosomes (10 ug protein for 1 h immediately prior to the cytotoxicity assay), and the percentages of cytotoxicity at the 20:1 E:T ratio were measured by flow cytometry.

NK-92, similar to primary activated NK (aNK) cells express high levels of NKG2D (Fig. 3a). In addition, NK-92 express TGFβRI and TGFβRII and CD95 (Fas). They do not express PD-1. Exosomes isolated from pre-ACT plasma of AML patients carry ligands for these receptors (Fig. 2a,b). Co-incubation of NK-92 cells with PKH67-labeled exosomes isolated from pre-therapy plasma significantly reduced expression levels of NKG2D on the NK-92 cell surface (Fig. 3b). Downregulation of NKG2D expression was measured by flow cytometry using exosomes isolated from plasma of four AML patients at diagnosis or from supernatants of leukemia cell lines, Kasumi-1 or ML-2 (Fig. 3c; Table 1). Also, exosomes isolated from plasma of 3 AML patients obtained pre-ACT or on day 7 or 21 after ACT similarly down-regulated NKG2D expression levels in NK-92 cells. The data for NKG2D expression after co-incubation of NK-92 cells of 4 AML patients are shown in Table 1. The data indicate that the mean % inhibition of NKG2D expression on NK-92 cells by AML exosomes was 40%. Interestingly, exosomes from post-ACT plasma (days 7 or 21) were less inhibitory than pre-ACT exosomes (Table 1).

**Table 1:** Inhibition of NKG2D expression levels by AML exosomes from pre-therapy or post-therapy plasma of AML patients treated with adoptive transfers of NK-92 cells.

Down-regulation of NKG2D expression on NK92 cells^a^							
		Pt 1	Pt 3	Pt 4	Mean	% INHIB	*p*-value
**At diagnosis** ^b^	%NKG2D^+^	47	55	57	53	39	<0.001
	MFI	6.1	4.8	6.7	5.8	54	<0.003
**Pre-ACT**	%NKG2D^+^	50	53	48	50	43	<0.001
	MFI	6.5	6.9	6.3	6.5	48	<0.003
**No EXO**	% NKG2D^+^	94	87	88	88		
	MFI	11.6	14.6	12.2	12.8		
		**Pt 3**	**Pt 4**	**Pt 6**	**Mean**	**% INHIB**	***p*** **-value**
**Post-ACT-d7**	%NKG2D^+^	84	85	80	83	11	<0.005
	MFI	9	8.8	9.2	9	27	<0.03
**Post-ACT-d21**	%NKG2D^+^	86	83	87	85	10	<0.01
	MFI	9.4	8.5	10	9.3	25	<0.04
**No EXO**	%NKG2D^+^	95	93	94	94		
	MFI	14	11	12	12.3		

^a^NK92 cells were co-incubated as described in Methods with exosomes isolated from plasma of AML patients enrolled in the phase 1 clinical trial. Exosomes were isolated from plasma collected just prior to ACT as well as on days 7 and 21 post-ACT. Percentages of NKG2D+ NK92 cells and mean fluorescence intensity (MFI) were measured by flow cytometry. NK92 incubated with medium but no exosomes served as controls.

^b^Exosomes were isolated from plasma of three different AML patients at the time of diagnosis. These patients were not enrolled in the ACT phase 1 trial.

Expression levels of NKG2D were reported to positively correlate with NK cell-mediated cytotoxicity^16^. Therefore, we expected NK-92 cell cytotoxicity to be reduced after co-incubation with AML exosomes. Cytotoxicity of NK-92 cells against K562 targets was indeed reduced from 70% to 12% by exosomes of pt #1 (Fig. 3d **)**. Also K562 cell lysis by exosomes from post-ACT plasma (d7) was inhibited (Fig. 3d). Exosomes isolated from pre-therapy plasma of 3/7 AML patients significantly reduced lysis of K562 targets (see Table 2). The addition of neutralizing anti-TGFβ Ab to the assay in part restored NK92 cytotoxicity from 20% to 52% (Fig. 3e). The data suggest that TGFβRs on NK-92 cells were engaged by AML exosomes in modulating NK-92 cell cytotoxicity. Importantly, exosomes from pre-ACT plasma of all 7 AML patients also inhibited lysis of NK-92 cells against primary AML blasts (Fig. 3f). The mean exosome-mediated suppression of NK-92 cell cytotoxicity against primary leukemic blasts was 37% with p < 0.002. The primary AML blasts were obtained from a leukapheresis product of an AML patient at diagnosis, and their phenotypic profile was determined by flow cytometry (Supplementary Figure 2) prior to use as targets in cytotoxicity assays.

**Table 2:** Inhibition of NK92 cytotoxicity against K562 cells by AML exosomes isolated from plasma of AML patients treated with adoptive transfers of NK-92 cells.

	% 7AAD^+^/total CFSE^+^K562 cells					
	Pt 1	Pt 3	Pt 4	Mean	% INHIB	*p*-value
Pre-ACT	12	17	9	12	78	<0.003
Post-ACT-d7	7	20	5	11	81	<0.005
No EXO	70	52	48	56		

In flow cytometry-based assays, NK92 cells served as effector cells at the E:T ratio of 20:1. CFSE-labeled K562 cells served as target cells.

### AML exosomes inhibit migration of NK-92 cells

**Figure 4:**
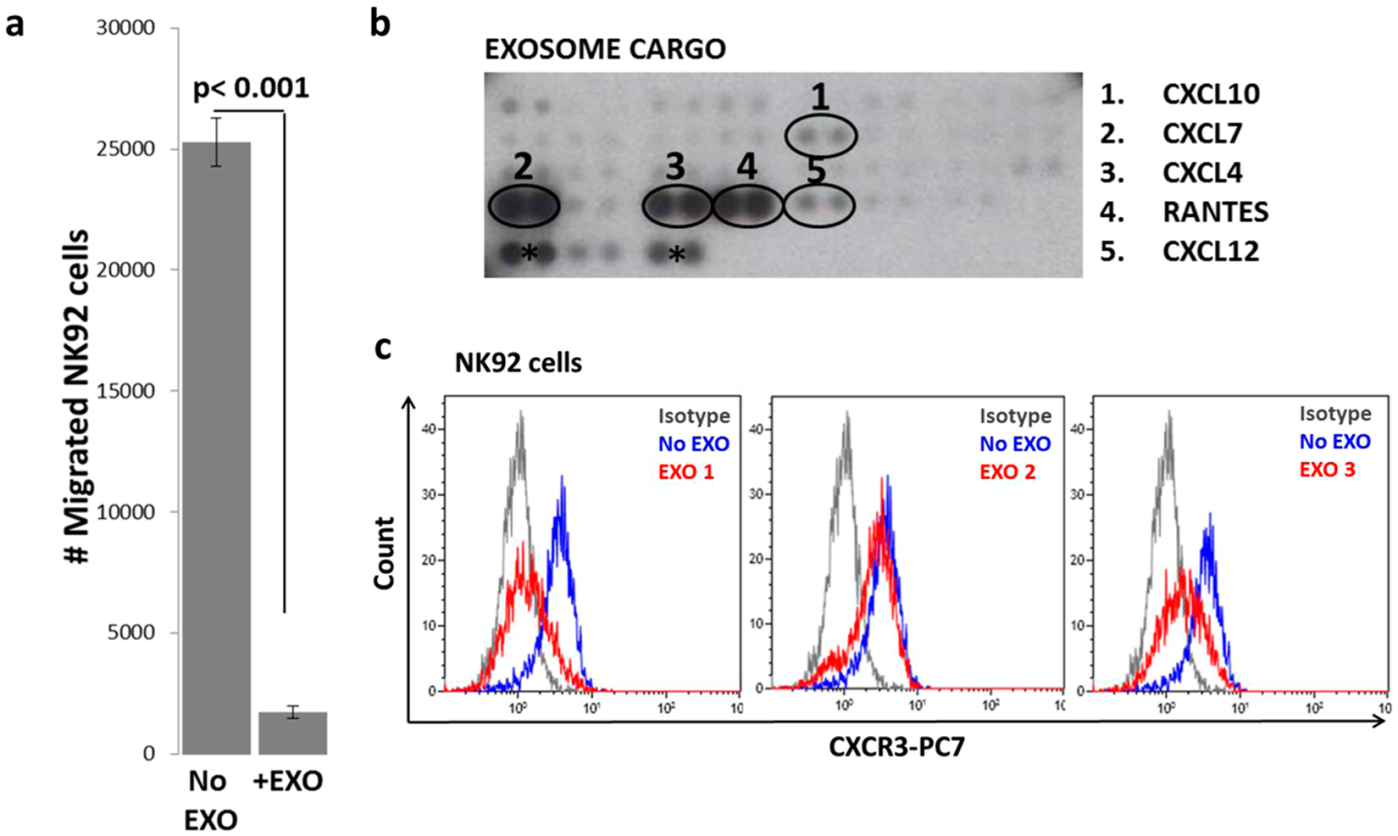
Migration of NK-92 cells toward leukemia cells is inhibited by AML exosomes. (**a**) AML exosomes inhibit NK-92 cell migration toward leukemia cell (Kasumi)-derived supernatants. NK-92 cells (1 × 10^6^) were pre-incubated ± exosomes from plasma of a refractory AML patient (5ug protein) for 1 h and then placed in a top Transwell insert and incubated for 4 h prior to counting cells that migrated. The experiment was repeated twice with different AML exosomes. (**b**) Chemokine immune array for plasma-derived AML exosomes. Exosomes (100 µg protein) were lysed with 1% NP40 lysis buffer before addition to the binding buffer for detection of individual chemokines. *Indicates reference spots for array alignment. (**c**) Downregulation of CXCR3 on NK-92 cells following co-incubation ± exosomes obtained from pre-ACT plasma of three different AML patients. The original image of the array can be found in the Supplementary Information.

NK-92 cells migration directed toward factors present in a pre-cleared leukemia cell (Kasumi) supernatant placed in the lower chamber of Transwell plates was significantly reduced (p < 0.001) by AML exosomes (Fig. 4a) These AML exosomes carried high levels of CXCL4, CXCL7, and CCL5 (RANTES) relative to the reference HD exosomes in antibody microarrays (Fig. 4b). Expression of CXCR3 on the surface of NK-92 cells was reduced following co-incubation with AML exosomes (Fig. 4c). Thus, ligand-mediated down-regulation of CXCR3 expression by AML exosomes levels was likely responsible for slower migration of NK-92 cells.

### AML exosome up-take by aNK and NK-92 cells

**Figure 5:**
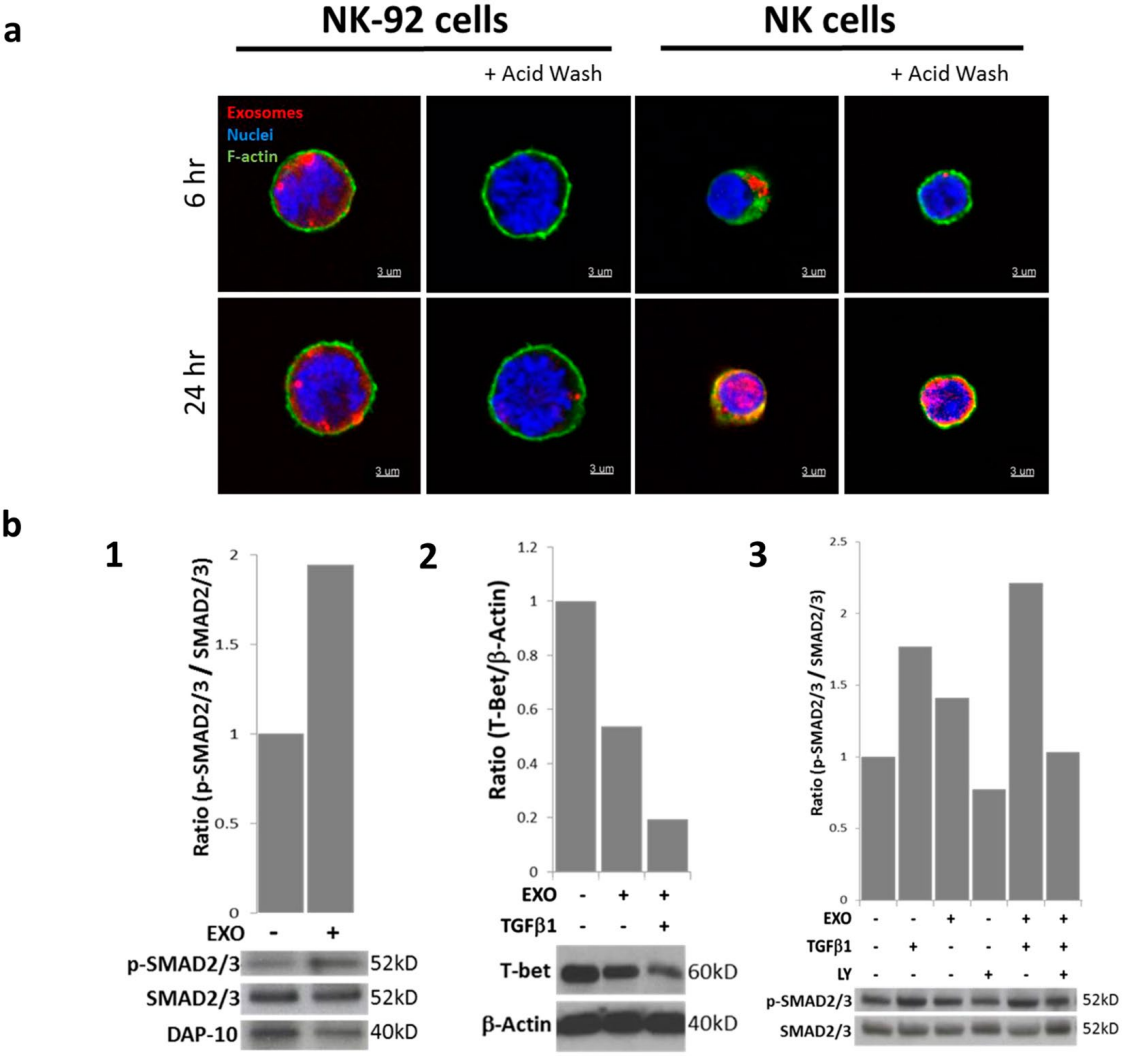
AML exosomes binding to in NK-92 cells. (**a**) NK92 cells do not internalize AML exosomes while primary aNK cells do. NK-92 or aNK cells (green) were co-incubated with PKH26-labeled exosomes (red) for different time periods as described in Methods. Confocal microscopy was performed to visualize labeled exosomes on the surface of NK-92 cells or inside aNK cells. Acid washes were used to strip exosomes bound to NK cell surface. Nuclei are stained blue. A representative experiment (1/3) was performed with different AML exosomes. (B.1) Semi-quantitative Western blot densities illustrating changes in SMAD2/3 phosphorylation and DAP10 expression in NK-92 cells co-incubated ± AML exosomes (10 ug for 24 h). (B.2) Suppression of T-bet in NK-92 cells co-incubated ± rhTGFβ1 (2 ng/mL), + exosomes (10 ug, from patient #3 and #4) or both for 24 h. (B.3) SMAD2/3 phosphorylation in activated primary NK cells was up-regulated after co-incubation (24 h) with rhTGFβ1(2 ng/mL) or AML exosomes (10 ug). It was downregulated to the baseline level when NK cells were pre-incubated (2 h at 37 °C) with LY2109761, an inhibitor of TGFβRI/II.

Since co-incubation of AML exosomes with NK-92 cells suppressed NK-cell functions, we expected that these exosomes are taken up by the recipient cells. However, confocal imaging of PKH26-labeled exosomes interacting with primary aNK cells or NK-92 cells showed that while the former readily internalized exosomes after only 2 h co-incubation, the latter did not internalize them even after 48 h of co-incubation (Fig. 5a). Following extensive washing of the recipient cells with acidic buffer to remove cell surface-bound exosomes, only primary aNK cells retained labeled exosomes in the cytoplasm (Fig. 5a). Labeled exosomes bound to the surface of NK-92 cells were removed by washing without any evidence for their presence in the cytoplasm (Fig. 5a). Similar results were obtained when we compared uptake of labeled exosomes by primary aNK vs NK-92 cells using Amnis flow cytometry (Supplementary Figure 3). These data indicate that NK-92 cells do not internalize AML exosomes.

### AML exosomes and TGFβ signaling in NK-92 cells

We showed that TGFβ carried by AML exosomes was responsible for downregulation of NK-92 cells cytotoxicity (Fig. 3e). As NK-92 cells expressed TGFβRI/II, we assumed that TGFβ^+^ exosomes signaled via these receptors, inhibiting cytotoxicity. Indeed, SMAD2/3 phosphorylation was increased while DAP-10 expression was decreased in NK-92 cells co-incubated with TGFβ+ AML exosomes (Fig. 5b1). AML exosomes alone or in combination with rhTGFβ1 downregulated expression of Tbet transcription factor in NK-92 cells (Fig. 5b2). Also, when TGFβ alone or AML exosomes alone were co-incubated with primary aNK cells, SMAD2/3 phosphorylation increased, but this increase was enhanced upon a combined treatment with TGFβ and AML exosomes. In the presence of LY2109761, an inhibitor of TGFβRI/II, SMAD 2/3 phosphorylation decreased to the baseline level (Fig. 5b3). These data suggest that TGFβ delivered to the surface of NK-92 cells by AML exosomes engages TGFβRI/II, upregulates SMAD 2/3 phosphorylation, downregulates DAP10 and further downstream, decreases Tbet expression levels, which translates into a loss in NKG2D expression levels and inhibition of cytolytic function in NK-92 cells.

### AML exosomes and PD-1 signaling in NK-92 cells

Exosomes from plasma of 3/7 AML patients carried PD-L1 (Fig. 2a). We, therefore, looked for PD-1expression on NK-92 cells and human primary aNK cells. Only few of the latter (~2% in HDs and ~ 5% in AML patients) were positive for PD-1 following IL-2 activation (data not shown). NK-92 were negative for PD-1, which rules out the participation of this pathway in suppression of NK-92 activity by PD-L1 + AML exosomes.

### AML exosomes up-regulate adenosine pathway in NK-92 cells

Adenosine (ADO) is a well-recognized inhibitor of immune cell functions^17^. The data in Supplementary Figure 4A show that growth of NK-92 cells was significantly inhibited in the presence of 2-chloroadenosine (CADO, a metabolically-stable analogue of adenosine). In immune cells, ADO signals via A_2A_R, up-regulates cAMP and inhibits cellular functions^17^. NK-92 cells are CD39+/CD73+ and express A_2A_R (Supplementary Figure 4B). NK-92 cells did not produce ADO in the presence of exogenous (e)ATP, although exosomes alone did, as shown in Supplementary Figure 4C and previously reported^18^. In the presence of (e)ATP and CD39+/CD73+ AML exosomes NK-92 produced high levels of ADO and its byproducts, inosine and hypoxanthine. Because NK-92 cells carry A_2A_Rs (Supplementary Figure 4B), it is likely that autocrine signaling of ADO or inosine binding to the A_2A_Rs expressed on NK-92 cells is responsible for a loss of functions in NK-92 cells.

### Effects of exosomes on cytokine production by NK-92 cells

NK-92 cells co-incubated ± AML exosomes were tested for levels of various cytokines using cytokine immunoblots or Luminex assays. No significant changes in levels of IL-1β, TNF-α, IL-8 or TGF-β1 were observed in immunoblots (Supplementary Figure 5). By Luminex, levels of IL-1RA, IL-10, IL-12, IL-2R, IP10 and IFN-γ produced by NK-92 cells co-incubated with exosomes remained unchanged. However, levels of CCL3 (MIP-1a), CCL4 (MIP-1b) and CCL5 (RANTES) were significantly reduced (Supplementary Figure 5). The data suggest that NK-92 cells exposed to AML exosomes have an altered cytokine production profile and produce significantly lower levels of several pro-inflammatory chemokines.

## Discussion

Adoptive cellular therapy (ACT) with various *in vitro* modified or engineered immune cells is rapidly emerging as a desirable experimental immune-based treatment for cancer. ACT is a potentially curable treatment for patients with cancer, especially for patients with hematological malignancies^2^. Patients with AML who are candidates for receiving ACTs generally had not responded to conventional therapies, experience drug resistance and have recurrent disease. From a therapeutic viewpoint, these patients represent an appropriate population for infusion of immune effector cells with potent anti-tumor activities. On the other hand, immune suppression and immune tumor escape are likely to be a significant barrier to the success of ACT in patients with advanced, refractory or drug-resistant malignancies. NK-92 cells selected for ACT are maintained as an IL-2 dependent cell line, proliferate rapidly *ex vivo*, are highly cytotoxic against leukemic targets and are resistant to apoptosis^19,20^. NK-92 cells are resistant to irradiation obligatory prior to their therapeutic use, remain viable and retain high levels of anti-leukemia cytolytic activity^8^. ACT with NK-92 cells in patients with relapsed/refractory AML was well tolerated and free of dose-limiting toxicity (DLT)^8^. Extensive immunological monitoring before, during and after ACT showed an absence of immune recovery and no long-term clinical responses. Levels of exosomes of immunosuppressive proteins were highly elevated in the patients’ plasma prior to ACT and remained elevated during and after therapy. These exosomes had profound inhibitory effects on anti-leukemia activity of transferred NK92 cells. They carried LAA and immunosuppressive proteins, including TGF-β and inhibited cytotoxicity, differentiation, proliferation and leukemia-directed migration of NK-92 cells used for therapy. AML exosomes did not induce apoptosis of NK-92 cells perhaps because they activated anti-apoptotic genes in these cells. AML exosomes induced phenotypic changes in NK-92 cells, including downregulation of an activating receptor, NKG2D or of CXCR4 involved in cell migration. The cytokine/chemokine production profile was altered in NK-92 cells co-incubated with AML exosomes, with a targeted loss in the levels of MIP1α (CCL3), MIP1β (CCL4) and RANTES (CCL5), the chemokines which modulate cell migration^21^. Production of soluble immuno-inhibitory factors, such as adenosine, inosine and hypoxanthine^22^ by NK-92 cells was significantly elevated upon co-incubation with AML exosomes, suggesting that autocrine inhibition of NK-92 functions via up-regulated A_2A_R contributed to their functional paralysis. Exosomes isolated from post-ACT plasma retained the immunosuppressive profile and the ability to down-regulate of NK-92 cells used for therapy. The NK-92 cells re-programmed by AML exosomes were viable but unable to migrate or mediate anti-leukemia cytotoxicity.

Reprogramming of NK-92 cell functions by AML exosomes did not require exosome uptake by recipient NK-92 cells. Although primary aNK cells internalize tumor-derived exosomes^23^, NK-92 cells failed to do so even after prolonged co-incubation with AML exosomes. PKH26 dye-labeled AML exosomes accumulated on the surface but not in the cytosol of NK-92 cells (Fig. 5a), while primary aNK cells rapidly internalized AML exosomes under the same experimental conditions. Thus, AML exosome-driven reprogramming of NK-92 cells was mediated through signals delivered to the cell surface. Among the signals delivered by AML exosomes that inhibited NK-92 cell cytotoxicity, TGFβ1 was an obvious candidate^13,14^. Neutralizing Abs specific for TGFβ in part reversed exosome-mediated inhibition of NK-92 cytotoxicity (Fig. 3e). AML exosomes enhanced TGFβ1 signaling via TGFβRI/II on the surface of primary aNK or NK-92 cells as evidenced by increased phosphorylation of SMAD 2/3 and its down-regulation by LY2109761, an inhibitor of TGFβ receptor signaling. TGFβ in the presence of AML exosomes downregulated expression levels of T-bet in NK-92 cells. These data suggest that AML exosomes re-program NK-92 cells using surface receptor-ligand signaling that translates into altered cellular responses.

**Figure 6:**
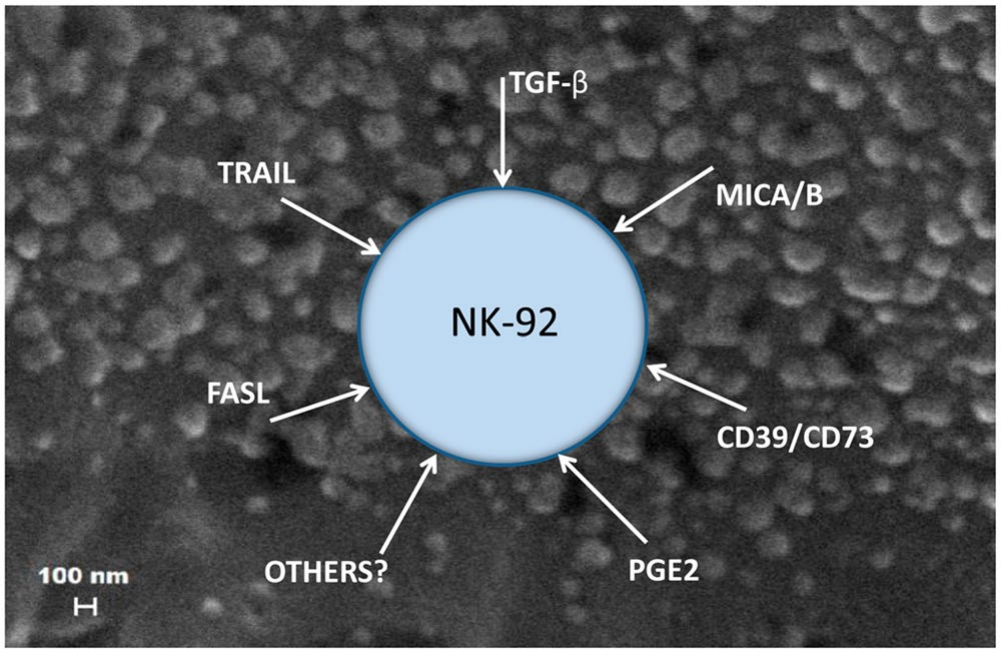
A hypothetical diagram of an NK-92 cell in the peripheral circulation of a patient with relapsed/refractory AML. The adoptively transferred NK-92 cell (similar to endogenous NK cells) is surrounded by masses of circulating exosomes, which carry immunosuppressive cargos, including various inhibitory ligands. Binding to cognate receptors on the NK-92 cell surface, these ligands, simultaneously activate multiple inhibitory pathways in the recipient cell. Exosome-driven negative signaling leads to a loss of NK92 cell functions.

In addition to TGFβ, AML exosomes carry multiple other inhibitory receptor/ligands and may deliver them as a “bundle” to the surface of immune or other recipient cells. NK-92 cells transferred into the circulation of AML patients are likely to encounter masses of circulating exosomes, all decorated with “bundles” of the immunosuppressive cargo (Fig. 6). These exosomes simultaneously deliver multiple inhibitory signals to recipient NK-92 cells, which upon translating these signals downstream become unable to mediate anti-leukemia activities. The molecular pathways engaged by exosome-mediated signaling are dependent on ligands in the exosome cargo and receptors expressed on the surface of the recipient cell. As NK-92 cells do not express PD-1, AML exosomes decorated by PD-L1 are unlikely to engage the PD-1/PD-L1 pathway in NK-92 recipient cells. Instead, the TGFβ/TGFβRI/II pathway, and other common molecular pathways, e.g., adenosine pathway, become activated by exosomes carrying cognate ligands and are responsible for downstream reprogramming. This type of reprogramming introduces a novel and so far underestimated mechanism of immune suppression existing in patients with advanced malignancies in the form of tumor-derived exosomes that may interfere with cellular adoptive therapies.

## Materials and Methods

### Patients and normal donors

Venous blood was collected from relapsed/refractory AML patients who were enrolled in the phase 1 clinical trial (NCT00900809) with a human, interleukin-2 (IL-2)-dependent NK-cell line, NK-92 expanded under cGMP conditions as recently described^8^. Seven patients completed the dose-escalating adoptive cell therapy (ACT). Six of the patients were male and one was female. The median age was 71 years (range 56–80 years). The trial and immunological as well as clinical results were recently described^8^. Plasma specimens were obtained from the patients enrolled in this clinical trial prior to and post therapy and at specified intervals during therapy and were banked. Plasma specimens were also obtained from additional AML patients seen at the UPMC Hillman Cancer Center at the time of first diagnosis (n = 10) and from AML patients (not enrolled in the phase 1 trial) with relapsed/refractory AML after conventional chemotherapy (n = 8). The plasma was collected and banked as per IRB-approved protocols (IRB #960279, #0403105 and #0506140). Specimens of plasma (n = 10) and peripheral blood mononuclear cells of healthy donors (HDs; n = 10) to be used for exosome isolation or to serve as exosome responder cells, respectively, were also collected. All patients and HDs signed the informed consent forms approved by the University of Pittsburgh Institutional Review Board. The methods used in this research were carried out in accordance with the IRB guidelines and regulations.

### Exosome isolation from plasma by mini size-exclusion chromatography (mini-SEC)

Exosomes were isolated from thawed, pre-cleared plasma samples as described by us earlier^15^. Size-exclusion chromatography (SEC) on Sepharose 2B columns was performed. The void volume fraction #4 contained the bulk of eluted exosomes. The eluted exosomes were characterized for the protein content, size, nanoparticle numbers, morphology, molecular profiles and suppressive functions as described^15^.

### Leukemia cell line-derived exosomes

Exosomes were isolated by the miniSEC method from supernatants of Kasumi-1 or ML-2 cell lines cultured in RPMI medium supplemented with 10% v/v ultracentrifuged FBS. Supernatants of confluent cultures were collected and exosomes in fraction #4 were harvested and were used as positive controls in some assays.

### Protein determination

Protein content and concentration of the isolated exosome fractions were determined using the Pierce BCA protein assay kit (Pierce Biotechnology, Rockford, lL, USA) following the manufacturer’s instructions. The protein concentrations are calculated as μg protein/1 mL pre-cleared plasma loaded onto the mini-SEC column.

### Exosome size and concentration assessment by tunable resistive pulse sensing (TRPS)

Size ranges and concentrations of isolated exosome fractions were measured using TRPS as recommended by the system manufacturer Izon (Cambridge, MA, USA). The measurement conditions and details were described by us earlier^15^.

### Transmission electron microscopy (TEM)

Transmission electron microscopy was performed as previously described at the Center for Biologic Imaging at the University of Pittsburgh^15^. Exosomes were visualized by the transmission electron microscope JEOL JEM-1011.

### Western blots

In preparation for Western blots, the exosome fraction #4 was concentrated by centrifugation on a 100 K Amicon Ultra 0.5 mL centrifugal filter (EMD Millipore, Billerica, MA, USA) at 5,000x g. Exosomes were tested for the presence of exosome markers, including TSG101 and other protein markers of interest as previously described^15^. PVDF membranes were incubated overnight at 4 °C with antibodies (Abs) purchased from various vendors and used at the dilutions described in the Supplementary Methods. Band intensities on exposed films were quantified using Image J software (NIH, USA). The integrated pixel value was determined for each protein band by multiplying image intensity and the band area after subtracting the mean background value.

### NK-cell culture

NK-92 cells for the *ex vivo* studies were obtained from the cGMP bank established by NantKwest and were cultured in X-VIVO 10 medium (Lonza, Wayne, PA, USA) supplemented with 5% (v/v) exosome-depleted human serum, 100U/ml penicillin, 100ug/ml streptomycin (Invitrogen, Grand Island, NY, USA) and 200 U/ml rIL-2 (Peprotech, Rocky Hill, NJ, USA). NK-92 cells were seeded at 1 × 10^5^ cells/mL and maintained in continuous culture at the cell density of 1 × 10^6^ cells/mL.

Primary human NK cells were isolated from the peripheral blood of normal donors by negative selection using AutoMACS (Miltenyi, San Diego, CA, USA)as previously described^23^. Isolated CD3-CD56+ NK cells were activated with IL-2 (100IU/mL, Peprotech) for 24–48 h prior to co-incubation with exosomes.

### Exosome labeling with PKH dyes and confocal microscopy

Exosomes for use in uptake experiments were labeled either with PKH26 (red fluorescence) or PHK67 (green fluorescence) and prepared for confocal microscopy as described in the Supplementary Methods.

### Functional studies with AML exosomes

Functional assays require substantial quantities of isolated plasma-derived and characterized exosomes. Changes in NK-92 functions following co-incubation with exosomes were measured using the exosomes isolated from pre-therapy plasma of the AML patients treated with ACT. As volumes of banked pre-therapy plasma were limited, we could not obtain sufficient quantity of exosomes from all patients for all functional assays and had to perform some experiments with exosomes isolated from plasma of relapsed/refractory AML patients who were not enrolled in the clinical trial. This is justifiable as exosomes from plasma of all relapsed/ refractory AML patients carry numerous inhibitory ligands and mediate suppression of immune cells *in vitro* ^13,14^. In all functional studies described below exosomes obtained from plasma of HDs and/or PBS (no exosomes) were used as “controls.” Functional studies performed with NK-92 cells co-incubated with AML exosomes are described in Supplementary Methods.

### Data analysis

Data were summarized by descriptive statistics (IBM SPSS, version 23) using means and standard errors (SE). Statistical analysis was performed using GraphPad Prism (version 6). As parametric tests, unpaired t test or one-way ANOVA and for non-parametric data, Mann-Whitney U or Kruskal-Wallis tests were used. Flow analyses were performed with Kaluza v1.5 (Beckman Coulter). A p-value of <0.05 was considered to be statistically significant.

### Data availability

All data generated or analyzed during this study are included in this published article and its supplementary information files. The details of the phase 1 clinical trial and the results generated in the course of this clinical trial are available from the corresponding author of this study.

## Electronic supplementary material

Supplementary Information
