# Supplementary material for: Circulating exosomes carrying an immunosuppressive cargo interfere with cellular immunotherapy in acute myeloid leukemia
Source: Sci Rep. 2017 Oct 31;7:14684. doi: 10.1038/s41598-017-14661-w (PMC5666018; doi:10.1038/s41598-017-14661-w)

**SUPPLEMENTARY FIGURES, SUPPLEMENTARY METHODS AND SUPPLEMENTARY DATA**

**Circulating exosomes carrying an immunosuppressive cargo interfere with cellular immunotherapy in acute myeloid leukemia**

Chang-Sook Hong, Priyanka Sharma, Saigopalakrishna S. Yerneni, Patricia Simms, Edwin K. Jackson, Theresa L. Whiteside, Michael Boyiadzis


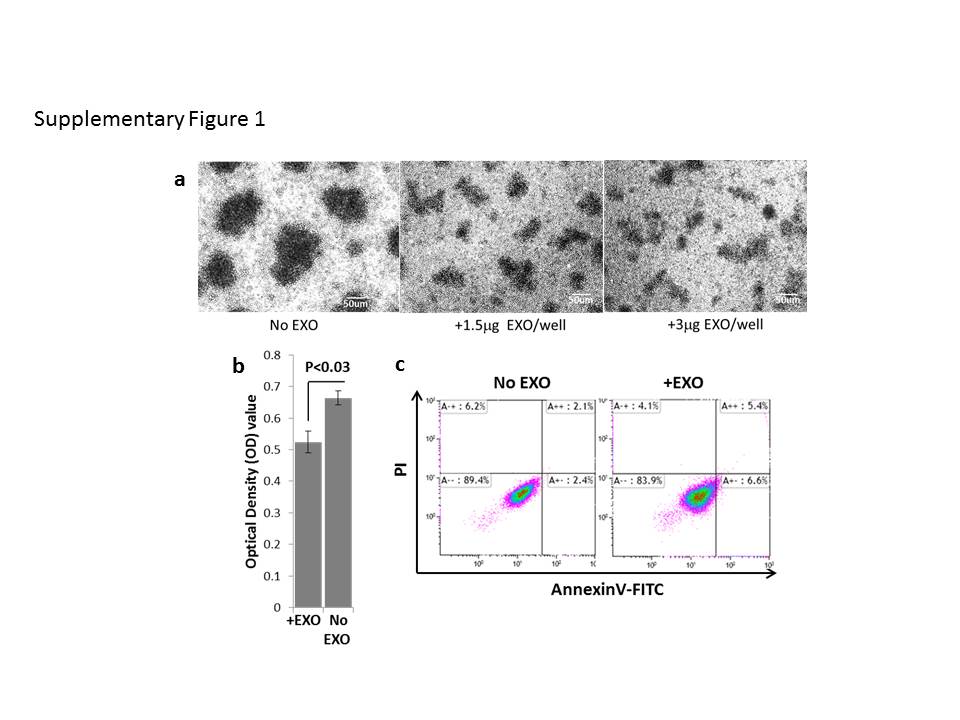


**Figure S1.** Effects of AML exosomes of proliferation and survival of cultured NK-92

cells. NK-92 cells were cultured in a 96-well plate as described in Methods ± AML exosomes for 20h. Note decreasing aggregation of cells when exosomes were added to the cultures. B. AML exosomes inhibited NK-92 proliferation as measured in MTT-based assays. C. Exosomes did not induce apoptosis in NK-92 cells. Representative flow cytometry for NK-92 cells co-incubated ± exosomes (10ug protein) for 24 h. The experiments were repeated 3 times with different AML exosomes.


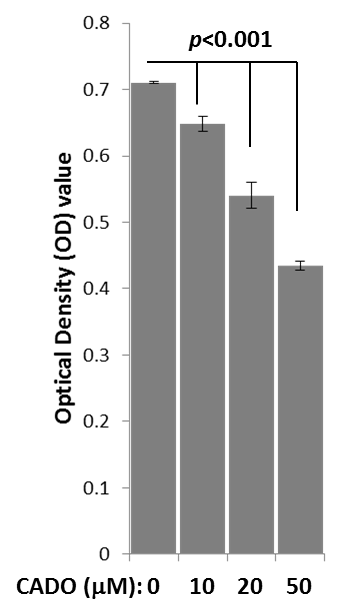

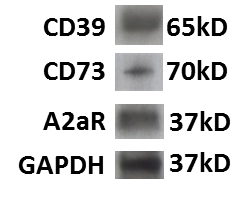

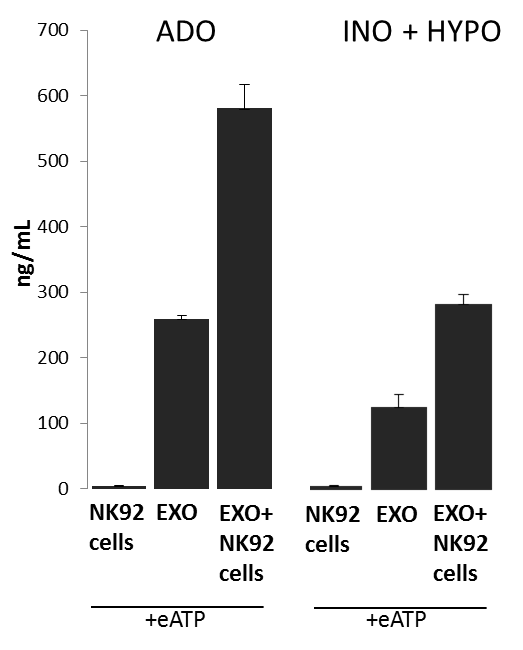


**a**

**b**

**c**

Supplementary Figure 4


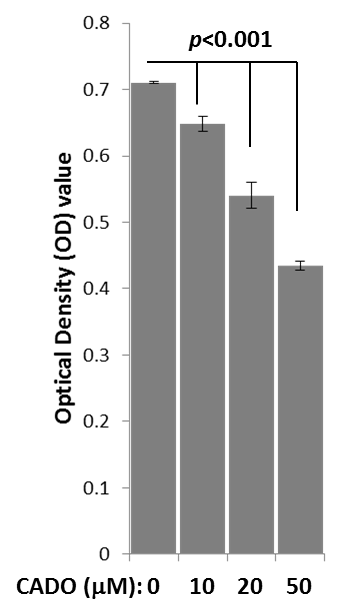

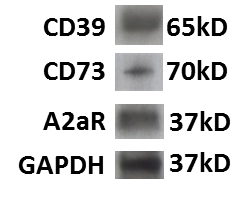

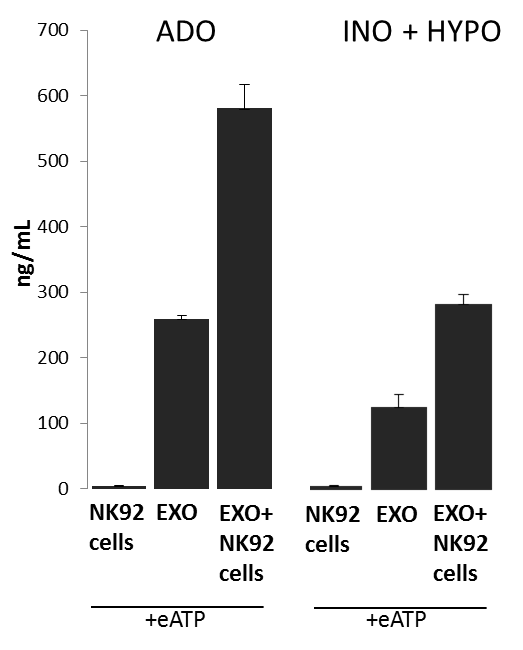


**a**

**b**

**c**

**Figure S4.**  **a**. Inhibition of NK-92 proliferation by CADO (2-chloroadenosine). NK-92 cells were incubated in medium as described in Methods ± CADO, and their proliferation was measured in MTT assays. **b**. Western blot of NK-92 cells shows that CD39, CD73 and A_2A_R are carried by these cells. **c**. Adenosine, inosine and hypoxanthine production by NK-92 cells co-incubated ± AML in the presence of exogenous (e) ATP and measured by mass spectrometry as described in Methods. Note that NK-92 cells + eATP make little or no adenosine or its byproducts.

Supplementary Figure 5


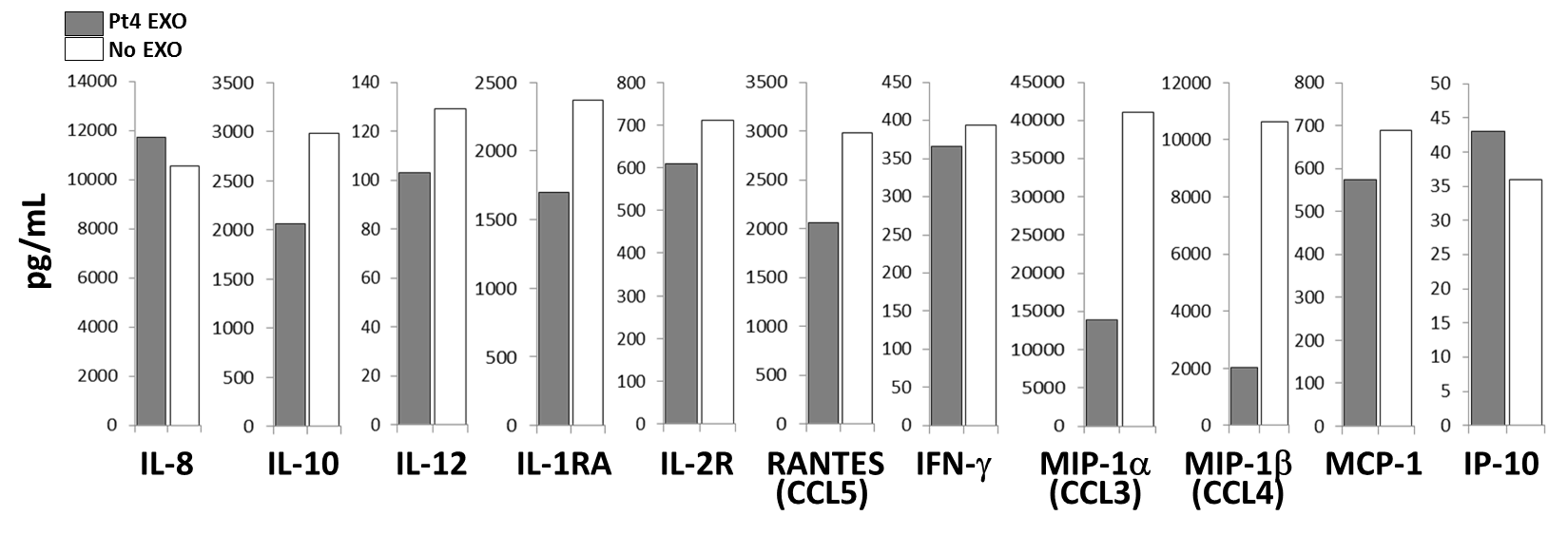


**b**


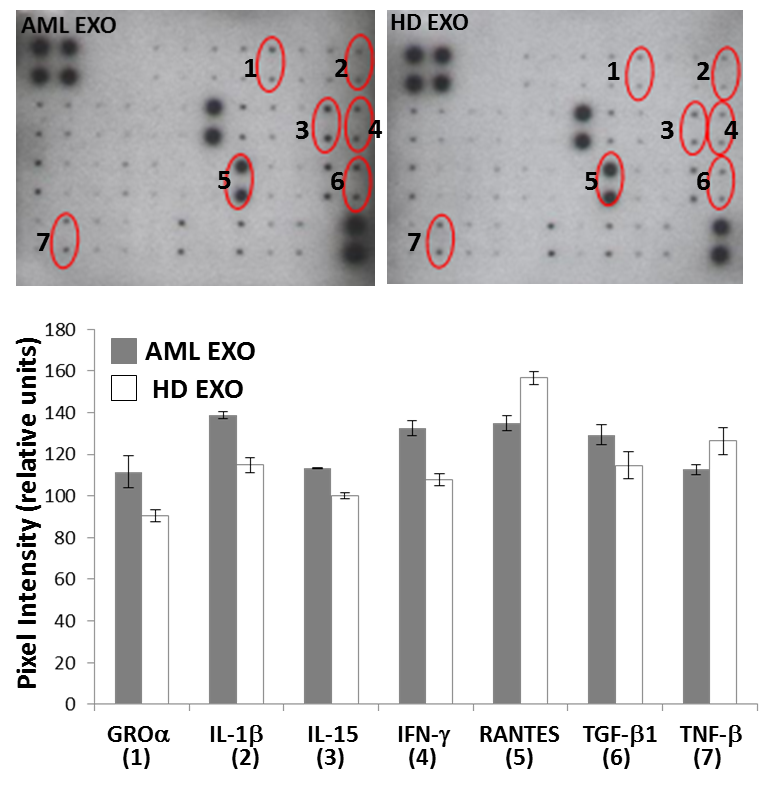


**a**

**Figure S5.** Effects of AML exosomes on cytokine/chemokine production by NK-92 cells. NK-92 cells (5 x 10^5^ ) were co incubated ± AML exosomes (20ug protein) from plasma of patient #4. Clarified supernatants of NK-92 were tested in Luminex (a) or immune blots assays (b) for cytokine/chemokine levels as described in Methods..

**SUPPLEMENTARY METHODS**

***Western Blot Reagents***

Abs from Abcam: CD34 (1:2000, ab81289), TSG101 (1:500, ab30871), CD96 (1:500, ab56653), Fas (1:1,000, ab1333619), A_2A_R (1:1000, ab3461); from Santa Cruz: CD39 (1:400, sc-33558), CD73 (1:400, sc-25603), PD-L1 (1:500, sc-19090), CXCR4 (1:500, sc-9046), GAPDH (1:500, sc-25778); from Thermo Fisher: CD123 (1:200, PA5-13582), from R&D systems: CLL-1 (1:2000, AF2946), PD-1(1:500, MAB1086); from Cell Signaling: TGF-β1 (1:1000, #3711), phosphor-Smad3(Ser423/425) (1:1000, 9520), Smad3 (1:1000, 9523), FasL (1:1000, 4273). Then, HRP-conjugated secondary antibody (1:3000 -1:5000, Pierce, Thermo Fisher) was added for 1h at room temperature (RT), and blots were developed with ECL detection reagents (GE Healthcare Biosciences).

***Flow cytometry***

Primary aNK and NK-92 cells were stained with the following mAbs: from EBioscience: CD39-PE, CD73-PC5, PD-1-PE, PDL-1, CXCR-PC7, CXCR3-PC7, CCR5-APC, Fas-PC7, CD56-APC, CD16-APC, NKG2D-PE; from Beckman Coulter: CD3-FITC or from R&D Systems: TGFβRII-PE. Matched isotype controls were purchased from the same companies. Following staining performed as previously described (24), cells were washed in PBS and examined in a Gallios flow cytometer (Beckman/Coulter).

***Immune arrays of exosomes***

5x10^5^ NK-92 cells were incubated + 20µg exosomes isolated from patients’ plasma for 24h. The supernatants were spun down for 10 min at 10,000 xg and filtered through 100 K Amicon Ultra 0.5 mL centrifugal filter (EMD Millipore, Billerica, MA, USA) to remove exosomes. The clarified supernatants were subjected for cytokines by Luminex (Human Cytokine Magnetic 30-Plex Panel, Thermo Scientific, Waltham, MA, USA) and also by cytokine antibody arrays (ab133997, Abcam, Cambridge, MA, USA) according to the manufacturer’s instructions. Densitometry data were obtained using Image J software (NIH, USA). To test for the presence of chemokines in the exosome lumen, 200µg exosomes were lysed with 1% NP40 lysis buffer with protease inhibitor cocktail (G6521, Promega, Madison, WI, USA). The lysate was tested for human chemokines using antibody arrays (ARY017, R&D Systems, Minneapolis, MN, USA) according to the manufacturer’s instructions.

***Exosome labeling and preparation for microscopy***

Exosomes (100 µg protein) were labeled either with PKH26 or PHK67 using fluorescent cell linker kits (Sigma-Aldrich) for 5 min. The staining reaction was stopped by adding 2mL of ultracentrifuged FBS. Labeled exosomes were washed with PBS using 100K Amicon Ultra 0.5mL centrifugal filter (EMD Millipore) and co-incubated with 2x10^5^ primary activated NK (aNK) or NK-92 cells in serum-free media for 90min, 3h, 6h, 18h, 24h and 48h at 37°C. To wash-off exosomes bound to the plasma membrane, cells were pelleted by centrifugation, re-suspended in stripping buffer (14.6 g NaCl, 2.5 ml acetic acid, 500 ml ddH_2_0) for 2min, washed 3x with PBS and fixed with freshly-prepared 1.6% paraformaldehyde (Electron Microscopy Services, Hatfield, PA) for 20 min at RT. Excess fixative was quenched by adding an equal volume of 1% BSA in PBS for 5 min followed by 3 x PBS washes. Fixed cells were cytospinned onto glass slides and permeabilized with 0.1% TritonX in PBS for 1min. To visualize F-actin and nuclei, cells were stained with Alexafluor488-Phallodin (1:40 in 1X PBS) or Hoechst 33342 (1:1000 in 1X PBS) purchased from Thermo Fisher Scientific, Waltham, MA, respectively. Imaging was performed using Carl Zeiss LSM 880 confocal microscope with fixed settings across all the experiment time points and the images were analyzed using ZEN Black software (Carl Zeiss Microscopy, Thornwood, NY).

***Exosome uptake by NK-92 cells measured by AMNIS image cytometry***

Following co-incubation with PKH26-labeled exosomes as described above, NK-92 or primary aNK cells were washed and stained with PE-labeled CD56 antibody (eBioscience). Image analysis was performed using an Amnis multispectral imaging flow cytometer (EMD Millipore, Billerica, MA, USA) as previously described (16) .

***Proliferation of NK-92 cells ± AML exosomes***

NK-92 cells (50,000/well) were co-incubated in wells of 96 well-plates + exosomes (10 µg protein) from plasma of relapsed AML patients for 24h at 37°C. Then, MTT solution (Sigma-Aldrich, St. Louis, MO, USA) was added and numbers of viable cells were measured according to manufacturer’s instruction.

***Apoptosis of NK-92 cells + AML exosomes***

Freshly isolated primary aNK cells were activated with IL-15 (20 ng/mL) and IL-2 (200 IU/mL) for 24 h. Both aNK cells and NK-92 cells (2x10^5^ /well) were co-incubated ± exosomes (10ug protein) for 24h. Apoptosis of NK and NK-92 cells was measured by flow cytometry using Annexin V-FITC Apoptosis Detection Kit (ab14085, Abcam, Cambridge, MA, USA).

***NKG2D down-regulation on NK-92 cells ± AML exosomes***

NK-92 cells were co-incubated with exosomes (10ug protein) for 24h at 37^o^C, stained with PE-conjugated anti-NKG2D Abs or isotype control Abs (Beckman Coulter, Atlanta, GA, USA) and analyzed for NKG2D expression levels using flow cytometry or confocal microscopy.

***Cytotoxicity assays***

Flow cytometry-based cytototoxicity assays were performed as described by us with minor modifications (25) using K562 cells as well as primary leukemic blasts isolated from patients with AML as target cells. NK-92 cells pre-treated or not with AML exosomes served as effector cells. Co-incubations of effector cells with exosomes were performed as described above. Following co-incubation, NK-92 cells were washed with PBS and then stained with the PHK26 dye as described above. Target cells were either K562 cells or primary leukemic blasts isolated from AML leukopheresis samples as previously described . Target cells were stained with 1.5 µM CFSE (Cell Trace, Life Technologies) according to manufacturer’s instruction. NK-92 cells and target cells were mixed at the 20:1 E:T ratio and incubated for 4 h at 37°C. Target cells were labeled with 7-AAD (BD Biosciences, San Jose, CA, USA) for 15 min to identify lysed cells and analyzed using flow cytometry. To block cytotoxicity of NK-92 targets mediated by TGF-β1^+^ exosomes, anti-TGF-β1^+^ antibody (1µg/mL, MAB240, R&D Systems, Minneapolis, MN, USA) or isotype control antibody was added to co-cultures of NK-92 exosomes.

***Transwell migration inhibition assay***

NK-92 cells (1x10^6^) were pre-incubated + exosomes isolated from relapsed AML patients’ plasma (5µg protein) for 1h at 37°C and then placed in the upper chamber of a Transwell plate (24-well plate, 5 µm pore, Corning, Lowell, MA, USA). A pre-clarified supernatant of a Kasumi cell line (from which exosomes were removed by previous ultracentrifugation at 100,000x g for 2h) was placed in the bottom chamber. The plate was incubated for additional 4h at 37°C. NK-92 cells that migrated to the bottom chamber were counted under a microscope.

***Mass spectrometry for adenosine and adenosine byproducts***

NK-92 cells (25,000) were co-incubated + exosomes (10µg protein) for 6 hrs in the presence or absence of 20 µM ATP. Supernatants were collected, spun down to remove cells, boiled for 5 min to inactivate adenosine-degrading enzymes and stored at -80°C for subsequent analyses. Purines were measured using liquid chromatography-tandem mass spectrometry as previously described (26).

**Supplementary Methods - References Cited**

13. Hong CS, Muller L, Boyiadzis M, Whiteside TL. Isolation and characterization of CD34+ blast-derived exosomes in acute myeloid leukemia. *PLoS One* 2014;**9**:e103310.

16. Muller L, Simms P, Hong CS, Nishimura MI, Jackson EK, Watkins SC, et al. Human tumor-derived exosomes (TEX) regulate Treg functions via cell surface signaling rather than uptake mechanisms. *OncoImmunology* 2016:in press.

24. Schuler PJ, Schilling B, Harasymczuk M, Hoffmann TK, Johnson J, Lang S, et al. Phenotypic and functional characteristics of CD4+ CD39+ FOXP3+ and CD4+ CD39+ FOXP3neg T-cell subsets in cancer patients. *Eur J Immunol* 2012;**42**:1876-85.

25. Kim GG, Donnenberg VS, Donnenberg AD, Gooding W, Whiteside TL. A novel multiparametric flow cytometry-based cytotoxicity assay simultaneously immunophenotypes effector cells: comparisons to a 4 h 51Cr-release assay. *J Immunol Methods* 2007;**325**:51-66.

26. Figueiro F, Muller L, Funk S, Jackson EK, Battastini AM, Whiteside TL. Phenotypic and functional characteristics of CD39high human regulatory B cells (Breg). *Oncoimmunology* 2016;**5**:e1082703.

**SUPPLEMENTARY DATA**

**
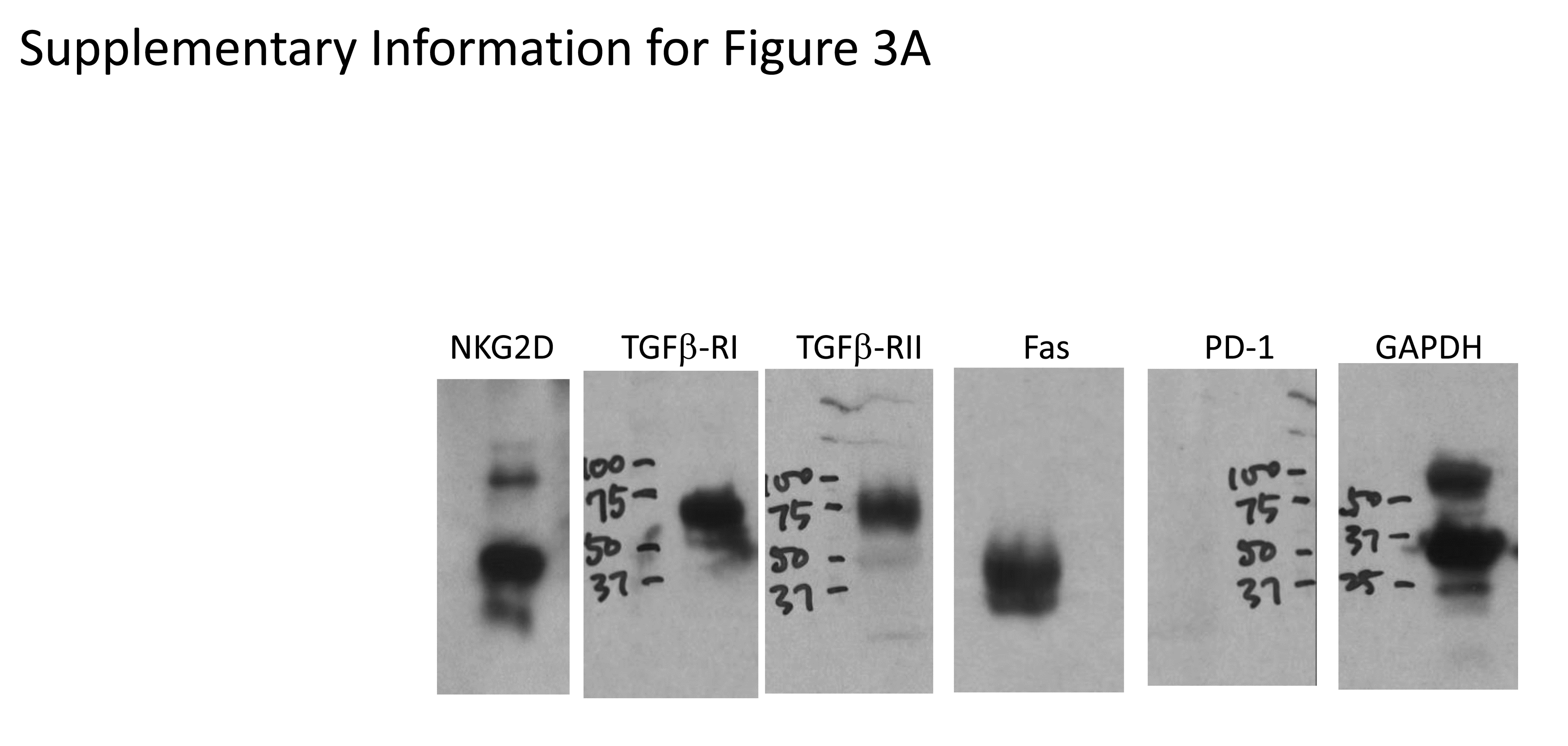
**


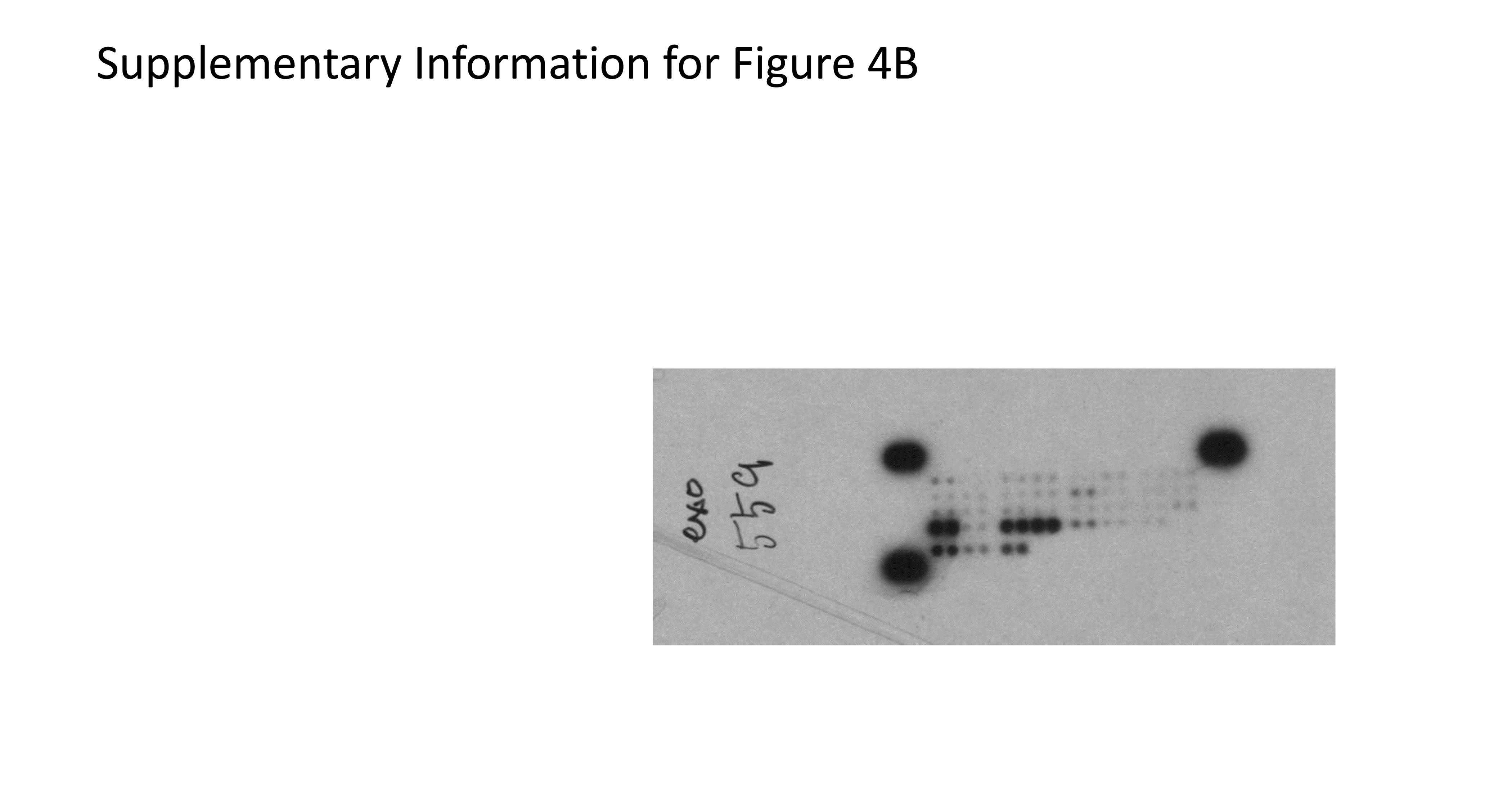

Supplement: Supplementary file 1 — Supplementary Information [file 41598_2017_14661_MOESM1_ESM.doc]
